# Should I stay or should I go? A qualitative study exploring participation in a urology clinical trial

**DOI:** 10.1007/s00192-018-3784-2

**Published:** 2018-10-17

**Authors:** Mabel Leng Sim Lie, Jan Lecouturier, Christopher Harding

**Affiliations:** 1 Institute of Cellular Medicine, Newcastle University, Newcastle upon Tyne, UK; 2 School of Geography, Politics and Sociology, Newcastle University, Newcastle upon Tyne, UK; 3 Institute of Health & Society, Newcastle University, Newcastle upon Tyne, UK; 4 Newcastle upon Tyne Hospitals NHS Foundation Trust, Newcastle upon Tyne, UK; 5 Freeman Hospital, Freeman Road, High Heaton, Newcastle upon Tyne, NE7 7DN UK

**Keywords:** Recurrent urinary tract infection, Clinical trials, Qualitative research methods, Recruitment

## Abstract

**Introduction and hypothesis:**

The aim of this study was to identify modifiable factors to improve recruitment in a urology clinical trial of women with recurrent urinary tract infection (rUTI). An embedded qualitative study was conducted with patients and recruiting clinicians in the first 8 months of the trial. We present a matrix of factors influencing how patients make decisions about trial participation.

**Methods:**

This was a qualitative study using telephone interviews. When they were first approached about the trial, women were asked to complete an expression of interest form if they wished to be contacted for an interview. Data were analysed thematically. NVivo 10 software (Qualitative data analysis software. 10th ed: QSR International Pty Ltd; 2012) was used as a management tool.

**Results:**

Thirty patients and 11 clinicians were interviewed. Influences on patient participation included the impact of rUTI on quality of life (QoL), understanding of antibiotic resistance, and previous experiences with antibiotics either positive or negative. Very few women who declined the trial agreed to be interviewed. However, some of those who participated had reservations about it. These included the perceived risk of trying a new treatment, trial length, and the burden of participating. One person interviewed left the trial because of repeated infections and difficulties getting general practitioner appointments.

**Conclusions:**

A combination of factors worked to influence women to decide to participate, to remain in, or to leave the trial. A better understanding of how these factors interact and work can assist in the recruitment and retention of individual trial participants.

## Introduction

Achieving patient recruitment and accrual targets is essential for the success of randomised controlled trials (RCTs), the current gold standard in clinical research [1]. Efforts to improve these factors have included trials on recruitment strategies [2] and interventions to improve informed consent [3]. A systematic review of strategies to improve trial recruitment highlighted the importance of raising patients’ understanding of their health problem and how it impacts them [2]. Qualitative interventions to improve informed consent have provided a means to understanding recruitment difficulties and how to overcome them [3]. As a result, the design of trial processes has undergone a number of improvements, such as inclusion of qualitative methods to identify and overcome barriers to recruitment [4] and, to keep pace with changing societal norms, to explore patient expectations and preferences, but further work is needed in this area.

Retaining participants in a clinical trial is another area of focused research [5, 6]. Zweben et al., for example, proposed a list of tailored routine and non-routine strategies to address the different risk levels of protocol adherence [5]. Some practical examples include obtaining permission to re-contact at a later time by sending strategic letters or e-mails. The importance of communication skills that engender ongoing trust has also been highlighted [6]. Underlying these attempts was the quest to understand decision-making processes about trial participation [7, 8], which were found to be influenced by the trial being directly relevant to individual patient circumstances, comfort and safety being carefully considered [7]. Other factors were strong individual treatment preferences for aspects of the trial, and these factors were often influenced by personality traits [8]. The social actors in this context are patients and the trial recruiter. The focus of the information being exchanged is in the context of trial design and process.

Addressing an identified gap in knowledge, research has been conducted into the experiences of trial staff [9, 10]. Specific research into the experiences of trial recruiters uncovered the need for more training, for example, in exploring treatment preferences in more depth rather than requiring participants to accept them at face value and closing off further discussion [11]. At the organisational level, there has been qualitative research with staff, investigating the barriers and facilitators that can take the form of norms, structures and processes present in the conduct of trials, and competitive pressures to perform well, resulting in inducements and enhanced care for patients [12, 13]. A review of the literature has raised the issue of over-emphasis on barriers to recruitment to the neglect of assessing facilitators, as found in successful trials [4]. The literature emphasises the need for more engagement of patients in understanding the trial and its processes [14–16]. Apart from the lack of patient-centred interaction in discussion of the trial [17], other barriers to recruitment relate to participant co-morbidities, particularly in older people; their impact on the practicalities of participating [18]; and the logistics of journey length [19]. In one approach to trial recruitment using patients’ risk–benefit assessments, patients’ decisions were context-dependent [20], especially if the benefit of close monitoring by specialists was opposed to their poor access to specialist services outside trial participation. The need for a more in-depth investigation of both decision making about UTI prophylaxis [21] and in recruitment into urological trials in primary care [7] is also highlighted.

We report on a qualitative study embedded in a clinical trial investigating the effectiveness of non-antibiotic prophylaxis for recurrent urinary tract infection (rUTI) in women. The aim of the embedded qualitative study was to inform the study team of potential barriers to recruitment and retention in the first 8 months of recruitment. This study adds to the knowledge around participant decision-making processes about prophylactic treatment and participation in urological trials.

Alternatives to Prophylactic Antibiotics for the Treatment of Recurrent Urinary Tract Infection in Women (ALTAR) is a multicentre, pragmatic, patient-randomised, non-inferiority trial comparing two treatments for preventing rUTI in women during a 12-month period of treatment and in the 6 months following treatment completion. Standard treatment is once-daily prophylactic antibiotic administration of licenced drugs recommended for this purpose: trimethoprim 100 mg, nitrofurantoin 50 or 100 mg or cefalexin 250 mg. The choice of antibiotic is determined by considering previous bacterial sensitivities, safety and patient or clinician preference. The alternative (experimental) treatment is 1 g per os twice daily of the urinary antiseptic methenamine hippurate (Hiprex) for 12 months. Participants in both arms would continue to receive treatment courses of antibiotic for UTI as needed.

The trial (including the embedded qualitative study) received a favourable ethical opinion from the North East - Tyne & Wear South Research Ethics Committee (Ref: 15/NE/0381). This research was funded by the UK National Institute of Health Research’s Health Technology Assessment Programme and was a commissioned call.

## Methods

### Recruitment to the qualitative study

Recruiting staff (trial nurses and doctors) introduced the qualitative study when women were approached about the ALTAR trial. All women who were eligible for the trial were asked to participate in the qualitative work, including those who had expressed a desire not to participate in the study. Recruiters explained the importance of understanding why people do and do not participate and how we sought to improve the way trials are conducted. Patients willing to be approached about the qualitative study were provided with a separate information sheet and contact form (expression of interest) in a reply-paid envelope to complete and return to the clinical trials unit (CTU). The CTU informed the qualitative team of all patients willing to be approached about an interview (including those who later left the trial). The aim was to conduct semi-structured interviews with patients within 2 weeks of the initial approach. Where possible, a second interview was conducted 3–6 months later to capture participants’ views on trial processes, outcome measures and reasons for leaving the trial. Clinicians [research nurses (RN) and principal investigators (PI)] were approached via e-mail a month after recruitment commenced at their site.

### Data collection

Patients who completed and returned a contact form were telephoned to answer any questions they may have about the qualitative study and to provide consent to participate; if they were happy to participate, a date and time was arranged for the interview. Most preferred to be interviewed when first contacted. Telephone interviews were conducted using topic guides developed with the input of the study team and Patient and Public Involvement group. Questions addressed participants’ experiences of trial recruitment and conduct, as well as acceptability of the intervention for both patient and clinician. Modifiable factors identified were noted immediately after the interview and fed back to the trial management group or chief investigator and CTU team. The interviews, prefaced by informed consent, were carried out by co-authors MLSL and JL, digitally recorded with the permission of the interviewee and transcribed verbatim.

### Analysis

Interview transcripts were checked and anonymised. Data was analysed drawing upon the constant comparative method, and a thematic coding frame was agreed upon between JL and MLSL. NVivo was used as a tool to manage and code transcript data, which was stored on password-protected drives. The overall headline results were made available to inform change in study procedures before the end of the first year of the recruitment phase.

## Results

### Participants

Between July 2016 and March 2017, 77 patients agreed to participate in the trial and were randomised. Eighteen were required to complete a washout period, as per protocol, as they had previously had prophylactic antibiotic treatment for rUTI. Thirty-five expression-of-interest forms were received for the qualitative study, and 30 participants were interviewed. One patient had already been approached about the trial but was deemed unsuitable because of confidential health reasons; as this person had some useful comments about the investigational medicinal products, their views are reported in this paper. Eleven RNs and two consultants (COs) were also interviewed. Eleven patients had a follow-up interview 3–6 months after the first interview (Table 1).

**Table 1:** Detailsof participants approached for the qualitative study

Patients and recruiters	
Trial participants	27^a^
Randomised to:	
Antibiotic	16
Methenamine	12
Second interview conducted	11^b^
Randomised to:	
Antibiotic	5
Methenamine	6
Interviewee unsuitable for trial	1
Interviewee declined trial^c^	1
Not contactable	5
Recruiting staff	
Research nurses	7
Consultants	2
Non-contactable	5

^a^Experienced an antibiotic washout period prior to trial entry - 1 patient

^b^Number of breakthrough infections recorded - 12

^c^Participant declined the trial was on Hiprex but stated she was willing to be contacted again, as she was very interested in the effects of the drug and the research we were conducting

The sample was diverse in that there was a wide range of ages—from teenage to 80+; 12 patients had partners, and five were single. Three patients discussed the trial with a health professional only; the remainder did so with their partners or family and friends. Experience of rUTI ranged from 2 years to a life-long chronic condition; many could be defined as “expert patients” (RN103). Only three had previously participated in research.

### Alerts to trial team

Issues identified that could impact recruitment and were resolvable included lack of clarity in the patient study information or consent process. The immediate feeding back of these issues enabled the chief investigator and trial team to rapidly resolve these issues. Some issues included whether adverse events (AEs) or severe adverse events (SAEs) reported by interviewees had been recorded in the trial data.

### Views of the trial

#### Themes

The interviews were driven by the need to explore factors around the trial processes and conduct: for example, views on trial information and consent process for both patients and recruiting staff. However, additional data was obtained on other themes identified, such as experience of rUTIs and their management and views of taking antibiotics.

#### Was the trial information comprehensive?

Patients recruited into the trial were provided with an information booklet covering the purpose of the study, the reason they were invited to participate, randomisation, the benefits and disadvantages or risks of taking part, the side effects of the drugs and patient confidentiality. Most patients were happy with the way information was conveyed to them verbally by the RN and appreciated the ongoing support they provided. The written information also provided reassurance and could be shared with family. However, some patients had queries about the actual workings of the trial drugs. In one of the first interviews, an interviewee raised a question about what was meant by an antiseptic as opposed to an antibiotic (this was reported to the Trial Management Group). These are common terms in the biomedical vocabulary that may be imprecise to a layperson, who then resorts to the Internet to find out how the medication works [Patient (PAT)1008], including how its mechanisms of action could be improved [Table 3, quotation 1 (Q1)]. It was asserted by clinicians that most patients were familiar with the idea of taking prophylaxis, commonly understood as a low dose of antibiotic to stave off infection. Nevertheless, the expectations of the treatments in question caused some questions to be raised about whether patients had a correct understanding about the trial drugs. Some appeared to believe that the trial would find the root problem and cure the condition (Q2-4). Patient beliefs and attitudes can contribute to the willingness to participate and stay in a trial [18]. However, it is important that they be based on an adequate understanding of the workings of the trial drugs. This was fed back to the trial team to improve the preliminary counselling process.

#### Why are patients willing participants?

From interviews with both patients and clinicians, there was agreement that the patient cohort were generally willing participants for a number of reasons, the most prevalent being the desperation to find a solution to their problems. One interviewee summed up the impact of rUTIs on normal activities in her daily life (Q5). Most interviewees were informed about antibiotic resistance, but their participation in the trial was described by clinician interviewees as clutching at straws (RN1301) – (Q6-7). For patients who had been recipients of healthcare for other conditions, there was also the desire to reciprocate. Others referred to the chance to help themselves and at the same time help others in the course of the research as fuelling their reasons to join the trial. Some patients were persuaded by the extra monitoring they would receive as trial participants (Q8) and that the course of treatment they received would be no different to standard care (Q9). Interestingly, women also referred to concerns about antibiotics. Although ultimately not eligible for the trial, one patient who had taken one of the trial medications as part of routine care and was told it was an alternative to an antibiotic had searched for information (Q10-11). Some had prior problems with certain antibiotics taken in the past (Q12). Others were aware that one could develop a resistance to antibiotics if taken for an extended period and appreciated the chance to try an alternative (Q13).

#### Why may patients have reservations about the trial?

The aim was to speak to up to 15 patients who had declined and 15 who dropped out of the trial to determine their reasons, yet the recruitment of such patients to the qualitative study was difficult. Despite the fact that 60 patients declined to participate in the trial, only one returned an expression-of-interest form to be interviewed. This patient declined the trial, preferring not to take antibiotics for an extended period (Q14). To explore reasons for non-participation, screening logs were analysed for patients who declined the study (Table 2) within the time frame during which the qualitative work was undertaken. These reasons were reinforced in the interviews with clinicians and in patient/participant reservations about the trial.

**Table 2:** Screening log data: reasons for declining the trial

Reasons specific to trial		Other	
Aspects of trial medications involved	4	No reason given	22
Blood tests	1	Not interested	6
Washout	7	Moving out of area	1
Extended follow-up period	4	Other health problems	5
Inability to adhere to trial protocol	2	Feels well	3

Also recorded on the screening log were four patients who could not be contacted and one who did not attend the appointment with the research nurse

Despite the low number of interviews with those who declined, data from patient and clinician interviews pointed to a number of reasons patients may not be willing to participate in the trial. One reason given by recruiting clinicians was that women were unwilling to try something new and different, especially if a particular treatment was working well for them or if they had no UTI at that time (Q15-16). This suggested that such women wanted to “let sleeping dogs lie” and were not willing to take unnecessary risks. Table 2 demonstrates a number of women declined to participate because of the 3-month washout period if already on prophylaxis. This view was reinforced in the clinician interviews (Q17-18) as a fear of causing further UTIs. Clinicians also cited practical difficulties for patients, which could include attending appointments, being non-compliant with the drug regime or had lives complicated by other on-going health conditions (Q19).

#### Why might patients consider dropping out?

During the qualitative study, eight patients withdrew from the trial. One patient in the antibiotic arm was interviewed just after withdrawal and gave the reason as being repeat infections and difficulties with general practitioner (GP) appointments. Another reason was an underlying preference for a particular treatment, even though at the time of recruitment randomisation was accepted by the particular research participant (Q20). In most interviews, relatives of patients appeared to express support for their decision to enter the trial. In contrast, one patient reported her family and friends were concerned about her being on antibiotics for a whole year, yet she understood the importance of the time needed to test the effectiveness of the drug being trialled. Treatment effect and trial length were other reasons given (Q21). This could combine with treatment preference to tip the balance for the participant to withdraw (Q22). Apart from some patients forgetting to take their trial medication, a number of patients reported difficulties taking the tablet due to its size and taste. One patient resorted to halving the tablet to make it more manageable, and: “to get rid of the taste, I would reward myself with something sweet afterwards” (patient 1009). Although no one left the trial specifically due to difficulty taking the tablets, it could potentially be a contributing factor to non-adherence to trial procedures and possibly leaving the trial.

Most requirements of the trial were not too different to the regular pathway for such patients. However, research burdens placed upon participants were identified that could accumulate during the course of the trial to discourage commitment to processes (Q23-24). One such burden, especially for the elderly and infirm or those with busy work schedules, was the effort of getting the sample to their GP (Q25) Table 3.

**Table 3:** Interview questions and quotations (Q) from interviewees

Questions and quotation number	Interviewee response
Was the trial information comprehensive?	
Q1	I just wondered whether, like, a pH test… once you know that you are on the Hiprex, whether a pH test might be useful to see how acidic your urine is……Because what I read was that a lot of doctors recommend taking a vitamin C tablet at the same time as the Hiprex - PAT1018
Q2	Some women have heard about being on prophylactic, some of them have been on prophylactics already. But obviously, they understand it. I mean, you’re not having to teach them what prophylactic means and things like that, they all understand it. - RN1301
Q3	I feel bad with it, it does affect your whole life. So I was more than happy to go for the trial and try and seek it out, for want of a better word. - PAT1101
Q4	…on my second visit, when I went to collect the medicine, they said...um...they told me that if I had another infection, to go and see my GP. But I thought that this treatment would stop them altogether, so I was bit concerned about that at the beginning - PAT1008
Why are patients willing participants?	
Q5	I think a lot of people do not understand a UTI, they just kind of think waterworks. …But when it’s something, you know, basic things like walking. Walking, sleeping, sitting at work, having a conversation with people, when you have got that level of discomfort, it’s difficult to just carry on as normal, to be honest. So yes, it really does affect your quality of life - PAT1005
Q6	I am at a stage now where I am actually prepared to try anything to try and rectify this, if possible - PAT1011
Q7	As I say, I am pulling my hair out with this repeating occurrence with the infection and that so if I can do anything more hopefully-helpful - PAT1101
Q8	So for me it was like, I would be getting medication anyway, so I can get the same medication and be slightly closer monitored for the problem I was having – PAT1009
Q9	I thought if I can be part of research and get treatment and research can be done at the same time to sort of help women that are going through the same thing, I thought it’s a positive thing.- PAT1013
Q10	I started taking these and I was a little bit concerned. So I actually got on the Internet and researched this and it actually says that it is an antibiotic. So I was a little bit confused. - PAT1005
Q11	Well, I suppose I was worried about, what is this that I’m putting in my system? What effect is it having on me? Because when you read it turns into formaldehyde you’re like, “Oh my god is that actually good for me or not, I don’t know. - PAT1005
Q12	Specifically in terms of the UTIs, I am already allergic to penicillin, so I can see the, you know, for me, the use of antibiotics is already quite limited. So I can see the benefit of trialling other ways, you know, for people like myself. - PAT1018
Q13	I’m kind of glad that I was put on the antiseptic rather than antibiotic just because I know that obviously prolonged use of antibiotics, you can build up resistance to it - PAT1013
Why may patients have reservations about the trial?	
Q14	I do not think it’s healthy to take antibiotics for 12 months, I do not think the effect on your body is a positive one…. I just, I did not want to take them, I did not want to be selected for the route of antibiotics for 12 months. - PAT1014
Q15	That it is a trial puts them off, just the fact that we are a trial or study. It makes them think they are trying something new or different - CO1102
Q16	I have had some ladies that have said that since they have been to the hospital they have not had any urinary tract infections. So they’re not wanting to take part in the study just now because they’re quite, you know, everything’s going well for them and they do not feel the need to do anything extra at the moment. - RN1301
Q17	They’re scared not to have their treatment because for them the prophylactic antibiotics may be working to a point. They don’t necessarily want to come off of them and not have treatment. - RN1101
Q18	.”…. I think a lot of them would be rather hesitant to take on, or to stop, because actually for the first time in ages, they have got something that’s working. - CO1303
Q19	For whatever reason, their lives are chaotic or they’re unwell. I mean, I have had quite a few women, there’s a couple of women that have been diagnosed with cancer. And they’re not wanting, they’re just like, “I can’t think about anything else right now.” RN1301
Why might patients consider dropping out?	
Q20	Interviewer: Would you have come off the trial if you were given the antibiotic, do you think? Patient: No, I don’t think I would have. I would have continued with it and given it a try but I think I would have been very open to, more open to saying, “Well, if this is not working I’m just coming off it,” whereas with this one I want to participate until the time is up if I can. – PAT1020:
Q21	One of my main concerns was that a year was a very long time to do a treatment……… my worry about the antibiotic was that maybe it was too long, and there might be some damage to the stomach lining. With such a heavy, it’s not heavy obviously, it’s a small dose but it’s for a long time. – PAT 1008
Q22	I thought if I get the antibacterial, which I would have gone through, but if it made life not much better, it would have seemed a long drag. The year would have been a long year . – PAT1016
Q23	One of the things that we have found for the ladies that have taken part in the study is trying to get their head around the system of sending back samples if they have a urinary tract infection. The process of the paperwork, the pods that they have to put the samples in, we have had a problem with that. I do not know whether the people declining do not want any additional hassle, additional visits, additional medication. - RN1302
Q24	And we have had one lady already come off study, because she thought it would be a burden as well, after having initially being recruited. - RN1002
Q25	The biggest inconvenience is getting it to the doctors…. because of the hours that I work, it impacts on my working life.... Because you have got to be there before a certain time so that the carrier can get it down to the hospital. – PAT1011FU

*Q* quote number, *PAT* patient,* RN* research nurse,* CO* consultant,* FU* follow-up

### A matrix to understanding trial participation

**Fig. 1:**
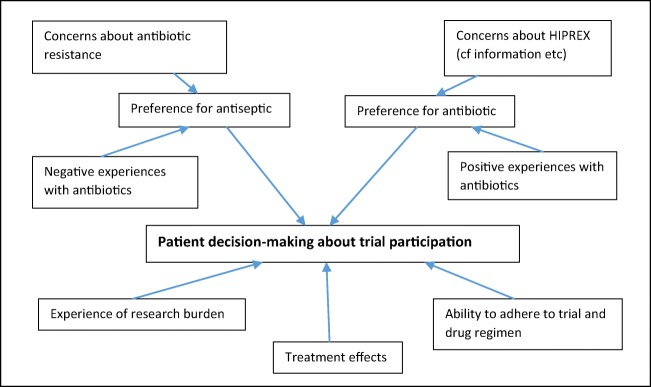
Matrix of factors for trial participation

These findings led us to devise a matrix (Fig. 1) to highlight and explain the interactions of influences on the decision-making process of women about continuing on this trial. While women with no real preference for either treatment are ideal candidates for a randomised controlled trial, there are many who may harbour a weak preference but are still willing to be randomised.

Those concerned about antibiotic resistance and/or who have had negative experiences taking antibiotics—for example, side effects like gut problems or thrush—may lean towards a slight preference for the antiseptic. If they experience a heavy research burden, for example, because of age, and/or a reduced ability to adhere to the trial and drug regime, the combined effect of these factors could likely push them to leave the trial prematurely. On the other hand, those who hold slight concerns about the new drug being tested and/or have had a good experience with antibiotics, especially as a prophylaxis, may hold a slight preference for antibiotics. Any problems arising from the research burden and/or ability to adhere to trial requirements could then nudge them in the direction of leaving the trial. Women with no preference for either treatment could still be influenced by these factors independently, which includes the effect of the treatment on their condition during the course of the trial.

## Discussion

The notion that RCTs offer the best means of assessing the clinical and cost effectiveness of medical interventions has been under scrutiny for a number of years. This is because the validity and reliability of RCTs are dependent on many human factors, in particular, behaviours of clinicians, patients and the organisational management involved in a trial [12, 13, 19]. The complex notions of randomisation and clinical equipoise can be difficult to negotiate between trial recruiter and potential participant [10, 22]. While the patient information sheet has detailed information about trial procedures, there will nonetheless be inadvertent gaps in understanding and retention on the part of the patient—for example, as expressed in their expectations of the trial. However, patients can still be highly motivated, even if these conditions exist, because of the impact and length of the condition on quality of life. Treatment preferences are complicated to ascertain [11]—for example, because of their variability—and it is important to recognise the distinction between a patient’s preferences and her decision to be randomised [23]. A patient may have a faint preference for participation but still agree to be randomised because of her desperation to seek out a suitable treatment. Altruism can come into play when deciding to join a trial, but practical concerns and difficulties can override all the good reasons for inclusion. The preference to not disrupt existing routines that currently work well is another reason for not being part of the trial, including not being subject to what is new and possibly risky and burdensome.

If concerns about taking an antibiotic prophylaxis or the antiseptic methenamine could be addressed by more information about how the two kinds of drugs work as prophylactic treatments and the risks involved, decision making about trial participation could be improved. The patient’s past experiences with antibiotics could form the basis for a more in-depth discussion about trial participation because of the way these have been identified as influencing the patient’s thinking about entering and staying in the trial. These findings resonate with other work reporting on the effect of past experiences of trial participation [24] and contextual factors unrelated to the trial itself [20].

Finally, this research provides a detailed insight into several aspects of the recruitment and retention process within a clinical trial involving female patients with recurrent UTI. It highlights several areas that future trials may want to consider when trial methodologies are designed. One aim of this research was to contribute to the literature on patient and clinician experiences of trial processes, and we identified some common themes: trial information, reasons for participation and reservations about taking part.

### Study limitations

For the 8-month duration of the embedded qualitative study, the ALTAR trial opened in five sites, a number of which did so towards the end of that period. The sample is thus skewed towards the first site to open. This impacted upon the ability of the qualitative study to have representation across the other four sites. We were unable to interview many patients who declined participation in or left the main trial. It is not clear whether this was due to an unwillingness to be interviewed or to site staff sometimes neglecting to mention the qualitative study. Nevertheless, we incorporated an interrogation of screening logs to gain any additional information from this patient group. Finally, inferences made from secondary sources (reports or reflections from clinicians) may not be as reliable as self-reported patient data.

## Conclusion

Trial recruitment, retention and research-study accrual are fundamental to the success of clinical trials. Our study contributes insights into the way factors identified in other studies come together to impact how patients decide to enter, stay or leave a trial.
